# When Do Employees Speak Up Under Job Stressors? Exploring the Potential U-Shaped Relationship Between Hindrance Stressors and Voice Behavior

**DOI:** 10.3389/fpsyg.2019.02336

**Published:** 2019-10-22

**Authors:** Longzhi Zhou, Kejian Yang, Zhen Wang, Zhengxue Luo

**Affiliations:** ^1^Air Force Medical University, Xi’an, China; ^2^School of Labor and Human Resources, Renmin University of China, Beijing, China

**Keywords:** hindrance stressors, challenge stressors, prohibitive voice, promotive voice, conservation of resources theory

## Abstract

Drawing upon the conservation of resources theory, we intend to examine the relationships between voice behaviors and job stressors. Specifically, we propose a non-linear relationship between hindrance stressors and prohibitive and promotive voice behaviors. Furthermore, we argue that challenge stressors moderate the non-linear relationship between hindrance stressors and voice behaviors. Based on a sample of 361 employees in China, our results indicate that the relationship between hindrance stressors and prohibitive and promotive voice is U-shaped. The relationships between challenge stressors and prohibitive and promotive voice are linearly positive. Moreover, challenge stressors moderate the relationships between hindrance stressors and voice behaviors; thus, when challenge stressors are high, hindrance stressors are negatively linear related to prohibitive and promotive voice behaviors, and when challenge stressors are low, hindrance stressors are curvilinearly related to prohibitive and promotive voice behaviors. The theoretical and practical implications of these results are discussed.

## Introduction

Employee voice refers to constructive change-oriented communication intended to improve the situation (LePine and Van Dyne, 2001). Voice behavior is beneficial for highlighting individual contributions (Kwon et al., 2016), improving team processes (LeClair-Smith et al., 2016), avoiding potential crises (Chamberlin et al., 2017), and enhancing organization performance (Hilverda et al., 2018). Job stressors have been regarded as one of the most critical inhibitors of voice behavior, since job stressors are widespread and unavoidable, more importantly; research has found that job stressors are negatively related to major work-related outcomes (Chen et al., 2017). Although literature has focused on the relationships between job stressors and voice behaviors (Hu et al., 2018), further research on psychological mechanisms and empirical evidence is still needed (Chen et al., 2015).

Recently, researchers have suggested that job stressors should be divided into challenge and hindrance stressors (Cavanaugh et al., 2000). Challenge stressors refer to job demands that present the potential for personal growth and rewards, such as workload, time pressure, and job responsibility. However, hindrance stressors refer to job demands that thwart personal growth and goal attainment, such as role ambiguity and role conflict (Abbas and Raja, 2018). Therefore, challenge stressors persuade employees to increase commitment with the expectation that employees will receive rewards or gains after they overcome the strain of stressors. Hence, challenge stressors are related to positive work attitude and voice behavior (Abbas and Raja, 2018; Ma et al., 2018).

**FIGURE 1:**
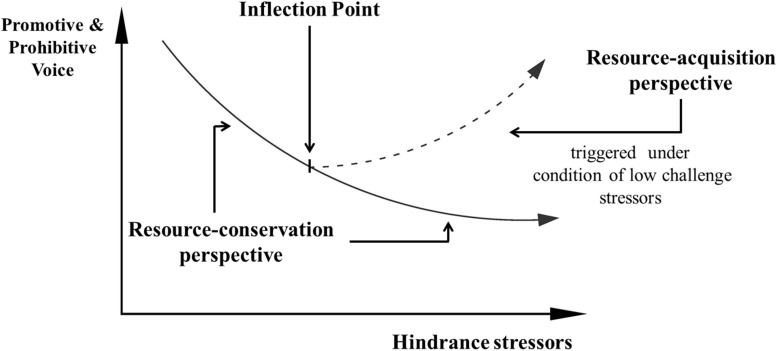
The relationship between hindrance stressors and voice: the resource-conservation perspective versus the resource-acquisition perspective triggered by different conditions of challenge stressors.

However, limited research has directly focused on the relationship between hindrance stressors and voice because there are two competing theoretical perspectives predicting this relationship. On the one hand, hindrance stressors discourage employees from enacting extra-role behavior and are negatively related to voice behavior (Yan and Xiao, 2016). According to the conservation of resources theory (the COR theory, Hobfoll, 1989), individuals are highly motivated to protect their limited resources, which drives employees to allocate more resources to deal with increased hindrance stressors (Lin et al., 2015), rather than wasting such resources on voice behavior (Abbas and Raja, 2018). On the other hand, hindrance stressors are positively related to voice behavior, as the expression of concern for harmful behavior can help to remove hindrance stressors (Ng and Feldman, 2012). The resource-acquisition motivation of the COR theory drives employees to speak up regarding the accumulation of additional resources from employers and support from others (Song et al., 2017). Therefore, the clarification of the hindrance stressors–voice relationship is valuable because both positive and negative associations between hindrance stressors and voice behavior are plausible. The possibility of both positive and negative relationships speaks to the potential for complex curvilinear relationships between hindrance stressors and voice behavior, a notion consistent with prior findings regarding the curvilinear relationship between voice and personal control (Tangirala and Ramanujam, 2008). Hence, we argue that the relationship between hindrance stressors and voice behavior is U-shaped (see Figure 1).

Furthermore, we intend to examine the combined effect of challenge stressors on the relationship between hindrance stressors and voice behavior, because employees rarely interface with isolated stressors and respond selectively to hindrance stressors or challenge stressors in practical work (Pearsall et al., 2009). However, limited attention has been paid to the combined effect of challenge stressors and hindrance stressors (Antwi et al., 2019). According to the challenge-hindrance framework, the positive appraisal of challenge stressors can partially offset the negative effects of hindrance stressors on voice behavior (Cavanaugh et al., 2000). Therefore, the positive expectation of potential gains from challenge stressors can decrease employee strain and resource losses, which results in employees being unable to reach a resource loss inflection point (Hollebeek and Haar, 2012). Employees continue to be driven by resource-reservation motivation at a considerably high level of hindrance stressors and exhibit a negatively linear relationship between hindrance stressors and voice behavior when challenge stressors are high. Thus, we believe that challenge stressors moderate the relationship between hindrance stressors and voice behavior.

Taken together, we intend to examine the U-shaped relationship between hindrance stressors and voice behavior by integrating the competing tenets of the COR theory. Moreover, we intend to explore the combined effect of challenge stressors on this U-shaped relationship. Then we elaborate on contributions in the discussion and suggest avenues for future research. By doing so, we can better understand employees’ voice behavior when they are confronted with challenge stressors and hindrance stressors, and our study provides theoretical suggestions and managerial implications for organizations.

### Theoretical Background

#### U-Shaped Relationship Between Hindrance Stressors and Voice Behavior

According to the COR theory (Hobfoll, 1989, 2002), we propose that individuals who are in stressful situations use voice behavior strategically as a means to protect or acquire resources. The COR theory assumes that individuals have limited personal resources, such as time, physical energy, and emotional energy. Individuals are highly motivated to protect their limited resources (resource-reservation motivation) and invest the necessary resources to acquire additional resource support or prevent resource losses (resource-acquisition motivation). Job stressors, especially hindrance stressors, refer to an appraisal process where individuals’ perceived demand exceeds their resources (Inzana et al., 1996), in which resource losses cause psychological strain and physical symptoms (Zhang et al., 2018). According to the COR theory, resource losses might decrease employees’ risky and costly voice behavior (resource-conservation motivation) (Yan and Xiao, 2016) or investment of resources to enact voice behavior for acquiring others’ support or additional resources (resource-acquisition motivation) (Tan et al., 2019). On the other hand, resource-acquisition motivation encourages employees to enact voice behavior for acquiring additional resources or stopping serious resource losses when they confront a high degree of hindrance stressors.

We propose that it is the degree of hindrance stressors that matters in regard to the fundamental change in resource motivation (see Figure 1). Moreover, there exists an inflection point that transforms resource-conservation motivation to resource-acquisition motivation. When hindrance stressors increase but are lower than the inflection point, employees are reluctant to enact voice behavior out of resource-reservation motivation or fears depleting valuable resources for the future (Wei et al., 2015). The COR theory also asserts that individuals are motivated to create situations that are pleasurable for themselves and avoid situations that might lead to the loss of any valued resources (Fatima et al., 2018). Resource-conservation motivation drives employees to preserve their valuable resources under hindrance stressors and not to enact risky voice behavior (Wei et al., 2015; Bolino and Grant, 2016; Yan and Xiao, 2016), because dealing with hindrance stressors is critical to prevent future resource losses (Schipani et al., 2018). Hence, hindrance stressors are negatively related to voice behavior. When employees reach an inflection point, the residual personal resources cannot withstand any further resource losses caused by hindrance stressors, and employees must turn to a resource acquisition approach such as voice behavior when resource losses reach a peak. At this point, the requirements for acquiring additional resources and demands to change the ongoing resource losses begin to outweigh the fear of resource losses (Qin et al., 2014). When the hindrance stressors increase past this inflection point, employees have to engage in a more innovative approach to change their status or speak up more frequently to stop undesirable behavior based on resource-acquisition motivation (Ng and Feldman, 2012). Qin has recently insisted that employees who are exhausted by hindrance stressors will increasingly engage in voice behavior because of resource-acquisition motivation (Qin et al., 2014). For example, individuals might use their voice to build new social relationships with individuals who can help them (Burris et al., 2017), impress their superiors with the aim of additional resource support (Tan et al., 2019), or target the undesirable behavior of colleagues or superiors to prevent resource losses (Frieder et al., 2015). Hence, high degrees of hindrance stressors are positive related to voice behavior. Therefore, the relationship between voice behavior and hindrance stressors is U-shaped.

In addition, following the prior research (Liang et al., 2012), voice behavior has been divided into two forms – promotive and prohibitive voice – because variations in the types of communicated messages can influence voice motivation (Kakkar et al., 2016). Promotive voice refers to the expression of new ideas, or innovations to improve organizational function, whereas prohibitive voice entails the expression of concerns regarding practices, incidents, or behaviors that are harmful to the organization. We argue that both promotive and prohibitive voice behaviors are closely related to hindrance stressors. When hindrance stressors begin to accumulate (but remain lower than the inflection point), employees choose to decrease any voice behavior under resource-reservation motivation (Knoll et al., 2016). When hindrance stressors accumulate above the inflection point, on the one hand, employees decide to put forward their concerns about existing problems that are harmful to the organization and call attention to resolving resource damage (prohibitive voice). On the other hand, employees use new suggestions or enact innovation to reverse the resource losses (promotive voice). Thus, we argue that a hindrance stressor’s inflection point exists and creates a curvilinear (U-shaped) relationship in which employees are likely to enact voice behavior when facing high or low levels of hindrance stressors but unlikely to do so at medium levels. Therefore, we hypothesize the following:

Hypothesis 1a: The relationship between hindrance stressors and prohibitive voice is U-shaped.
Hypothesis 1b: The relationship between hindrance stressors and promotive voice is U-shaped.

#### Combined Effect of Challenge Stressors and Hindrance Stressors on Voice Behavior

Although as kinds of job demands, both stressors cause strain and physical symptoms (Ma et al., 2018), challenge stressors focus on job demands that provide potential gains or future growth (Yang et al., 2017; Charkhabi, 2018). Then challenge stressors encourage employees to overcome strain and increase commitment in their work (Sun et al., 2018). Specifically, in contrast to hindrance stressors, which only thwart employee development and goals, positive expectation of challenge stressors typically evokes positive work attitudes and job performance (Lin et al., 2015). Moreover, individuals who exert positive appraisal positively commit in extra-role behavior reciprocally, such as organizational citizenship behavior (OCB) and voice behavior (Liu and Li, 2018). The higher the level of challenge stressors is, the more fulfillment employees feel and the more sufficient the resources for employees to engage in voice behavior (Chen et al., 2015). Specifically, employees who are confronted with challenge stressors choose to express concern for existing problems and call for an immediate solution to the problem (prohibitive voice). On the other hand, employees who encounter challenge stressors are motivated to enact more new ideas and suggestions to change the *status quo* (promotive voice). Therefore, we hypothesize the following:

Hypothesis 2a: Challenge stressors are positively related to prohibitive voice.
Hypothesis 2b: Challenge stressors are positively related to promotive voice.

We argue that the relationship between hindrance stressors and voice behavior may be better understood by examining the combined effects of challenge stressors according to previous research (Boswell et al., 2004). We feel that this is the most interesting combination because positive and negative stressors may occur simultaneously, but the potential influence of one type of stressor interacting with the other factors has been overlooked (Folkman and Lazarus, 1985). We propose that challenge stressors might alleviate employees from reaching the inflection point of the hindrance stressors and that employees are unlikely to speak up for resource-reservation motivation. According to the challenge-hindrance framework, the positive motivational effects of challenge stressors partially buffer the negative effects of hindrance stressors on extra-role behavior (Cavanaugh et al., 2000). Moreover, the interaction of the challenge-hindrance framework alleviates their negative effect on job satisfaction, commitment, and perceived organizational support (Hollebeek and Haar, 2012). In addition, particularly, the resources gathered when job demands are very challenging will be used to persistently deal with high hindrance stressors (Bakker and Demerouti, 2018). Research among Finnish teachers and dentists (Bakker et al., 2007) has shown that job resources are most predictive of work engagement when challenging job demands are high. In addition, the hindrance stressors contribute to fewer strains and physical symptoms when employees perceive psychological support presented by challenge stressors (Dawson et al., 2016). These results are consistent with the assertion that positive aspects might allow the employee to “thrive in the face of risk” (Gareis et al., 2009), indicating that the positive appraisal of challenge stressors will alleviate the negative effect of hindrance stressors. Hence, the positive effect of challenge stressors will prevent employees from reaching the resource exhaustion inflection point caused by hindrance stressors. Therefore, the relationship between hindrance stressors and voice behavior remains negatively linear past the inflection point. Specifically, on the one hand, when challenge stressors are high, employees still don’t want to express the existing problems (prohibitive voice) and enact new suggestion or innovation (promotive voice) for resource-reservation motivation. On the other hand, when challenge stressors are low, employees still are motivated to speak up for a high level of hindrance stressors for resource-acquisition motivation. Therefore, when challenge stressors are low, it leads to out the U-shaped relationship between hindrance stressors and voice behavior. Therefore, we hypothesize the following:

Hypothesis 3a: Challenge stressors would moderate the relationship between hindrance stressors and prohibitive voice such that the relationship is (i) linearly negative when challenge stressors are high but (ii) U-shaped when challenge stressors are low.
Hypothesis 3b: challenge stressors moderate the relationship between hindrance stressors and promotive voice such that the relationship is (i) linearly negative when challenge stressors are high but (ii) U-shaped when challenge stressors are low.

## Materials and Methods

### Participants and Procedures

The present study was based on the data collected from employees of an electronic manufacturing enterprise in Shaanxi, China. These employees were working in different departments, such as marketing, finance, production, human resources, and quality control. This company was encouraging employees to present constructive suggestions that could promote the development of the organization to address fierce competition. Because there was only one data source collected from employees, we took measures to control for the common method bias (CMB, Podsakoff et al., 2003), including different Likert scale formats, and we distributed survey questionnaires at two different points in time. We reminded the employees that the questionnaires were anonymous questionnaires, and included some interference in the items. The questionnaires were administered in the company’s conference room during work hours, the work stressors’ level was investigated at the first time point, and voice behavior level was investigated at the second time point. Prior to the beginning of the questionnaire, we assured participants of full confidentiality. All employees were coded and informed only to write their codes. Then we assigned a questionnaire to participants, and all completed questionnaires were returned directly to the researchers. The data were recorded and analyzed twice by two other researchers.

A total of 450 questionnaires were distributed each time, with 388 returned the first time and 373 returned the second time. A total of 361 questionnaires were ultimately matched and valid, giving a response rate of 80.22%. According to company records, the dropped responses were not significantly different from the valid responses in demographic characteristics: age (*t* = 1.31, *p* = 0.19), tenure (*t* = 1.1, *p* = 0.27), gender (Mann–Whitney *U*-test, *p* = 0.38), marriage (Mann–Whitney *U*-test, *p* = 0.12), and education (Mann–Whitney *U*-test, *p* = 0.09). The population characteristics of the sample were as follows: of the participants who completed the questionnaires, 200 were male (55.4%), and 161 were female (44.6%). The means and standard deviations of age and tenure were 34.47 (*SD* = 8.75) and 12.98 years (*SD* = 12.34), respectively. On average, 29.10% of respondents were unmarried (*N* = 105). Of the sample, 61.78% had completed college or post-high school education (*N* = 223).

### Measures

While all the measurement scales were originally developed in English, they were later translated into Chinese and applied in the Chinese context according to back-translation procedures (Brislin, 1980).

### Hindrance and Challenge Stressors

Hindrance and challenge stressors were measured using an 11-term questionnaire (e.g., “It is not clear to me what is expected of me on the job” and “I have a considerable amount of projects and assignments to accomplish”) developed by Cavanaugh et al. (2000). This measure consisted of five hindrance-related items and six challenge-related items. The response format used by Cavanaugh et al. (2000) ranged from 1, “produces no stress,” to 5, “produces a great deal of stress.”

### Voice Behavior

Voice behavior was measured using a 10-item questionnaire (e.g., “Speak up honestly with problems that might cause serious loss to the work unit, even when/though dissenting opinions exist” and “Proactively develop and make suggestions for issues that may influence the unit”) developed by Liang et al. (2012) and consisted of five promotive voice-related items and five prohibitive voice items. The response format ranged from 1, “strongly disagree,” to 7, “strongly agree.”

### Control Variables

Consistent with a previous study, we measured gender, age, tenure, marriage, and education because of their potential impact on voice behavior (Spector and Brannick, 2011). For example, highly educated employees were likely to have more ideas to voice than less educated employees (Liang et al., 2012), and experienced employees, reflected by age and tenure, showed more inclination to voice ideas than less experienced employees (Tangirala and Ramanujam, 2008).

### Data Analysis

We conducted hierarchical linear modeling (HLM) to examine our hypotheses using SPSS 23.0 and Mplus 7.0. The interclass correlation coefficient (ICC) (1) and ICC (2) are 0.015 and 0.225 for prohibitive voice, 0.068 and 0.581 for promotive voice, 0.073 and 0.607 for challenge stressors, and 0.041 and 0.358 for hindrance stressors, respectively. All the predictor variables are standardized to decrease multicollinearity (Dawson and Richter, 2006). According to Hayes’s (2013) and Dawson’s (2014) suggestion, we used multilevel, random-intercept, regression analysis to test the moderation effect of challenge stressors (M) on the U-shaped relationship between hindrance stressors (X) and voice behavior (Y) (O’Malley et al., 2015) at:

Yij=a+1jaM2+ijaX3+ijaXsq4+ijaXM5ij+aXsqM6+ije            ij(1)

a=1jr+10uj1

The regression equation (Equation 1) includes a quadratic element (Xsq), within-level residual variance (e_*ij*_), and between-level variance (u_1__*j*_). The voice behavior of employee i in group j was treated as the outcome variable in a two-level regression. Then, in order to distinguish whether there is a significant (linear or curvilinear) relationship at that a particular value of M, we start by rearranging Equation (1) above:

Yij=a+1jaZ2+ij(a+3aM5)ijXij+(a+4aM6)ijXsq+ije      ij(2)

a=1jr+10uj1

If there were a quadratic relationship, the value of (a_4_ + a_6_M) would be non-zero. In this situation, we would standardize the moderator at the value at low (*M* = -1) and high (*M* = 1) levels of challenge stressors to test whether the (linear or curvilinear) relationship is significant (Aiken and West, 1991). This was accomplished by parameter bootstrap estimation with Mplus to test the slope or curvature of relationship between hindrance stressors and voice behavior.

Confirmatory factor analysis (CFA) was conducted to test whether the four studied variables were distinct. The fit indices of the hypothesized factor model were compared with those of alternative factor models to confirm which better fit the data (Mathieu and Farr, 1991). According to a previous study (Joreskog, 1977), we held that the criteria of model fit are χ^2^/*df* < 5, standardized root mean square residual (SRMR) < 0.08, root mean square error of approximation (RMSEA) < 0.08, comparative fit index (CFI) > 0.9, and tucker-lewis index (TLI) > 0.9.

## Results

### Confirmatory Factor Analysis

First, we conducted a CFA to examine the discriminant validity of the study variables. The results of the tests of competing CFA models are shown in Table 1. We built a four-factor CFA (hindrance stressors, challenge stressors, promotive voice, and prohibitive voice) model to provide a the better fit to the data (akaike information criterion (AIC) = 20516.78, χ*^2^* = 478.26, *df* = 183, *SRMR* = 0.06, *RMSEA* = 0.07, *CFI* = 0.94, *TLI* = 0.94 for the total sample), compared with the alternative three-factor model (promotive voice and prohibitive voice were combined) [AIC = 20728.61, Δχ^2^(3) = 217.84, *p* < 0.01], as well as the other three-factor model (hindrance stressors and challenge stressors were combined) [AIC = 21240.35, Δχ^2^(3) = 729.58, *p* < 0.01]. The four-factor measurement model also fit better to the data than the two-factor and one-factor models (Table 2). The average variance extracted (AVE) and composite reliability (CR) are 0.53 and 0.85 for prohibitive voice, 0.61 and 0.88 for promotive voice, 0.60 and 0.90 for challenge stressors, and 0.61 and 0.89 for hindrance stressors, respectively. The results showed that the AVEs of all variants are above 0.5 and that the CRs of all variants are above 0.8, which confirmed acceptable construct validity (Hair et al., 2010).

**TABLE 1:** Confirmatory factor analysis.

**Model**	**AIC**	**χ^2^**	**df**	**χ^2^/df**	**RMSEA**	**CFI**	**TLI**	**SRMR**	**Δχ^2^(Δdf)**
4-factor (VB1; VB2; HS; CS)	20,516.78	478.26	183	2.61	0.07	0.94	0.94	0.06	
3-factor (VB1 + VB2; HS; CS)	20,728.61	696.10	186	3.74	0.09	0.90	0.89	0.07	217.84(3)
3-factor (VB1; VB2; HS + CS)	21,240.35	1,207.84	186	6.49	0.12	0.80	0.78	0.08	729.58(3)
2-factor (VB1 + VB2; HS + CS)	21,452.67	1,424.15	188	7.58	0.14	0.76	0.73	0.09	945.89(5)
1-factor (VB1 + VB2 + HS + CS)	23,626.31	3,599.80	189	19.05	0.22	0.34	0.27	0.26	3,081.54(6)

*VB1, prohibitive voice behavior; VB2, promotive voice behavior; HS, hindrance stressors; CS, challenge stressors; AIC, akaike information criterion; SRMR, standardized root mean square residual; RMSEA, root mean square error of approximation; CFI, comparative fit index; TLI, tucker-lewis index.*

**TABLE 2:** Means, standard deviations, correlations, and reliabilities.

	**Mean**	***SD***	**1**	**2**	**3**	**4**	**5**	**6**	**7**	**8**	**9**
1. Gender^a^	0.55	0.58									
2. Age	34.47	8.75	**-0.06**								
3. Tenure	12.98	12.34	**-0.14^∗^**	0.55^∗∗^							
4. Marriage^b^	1.75	0.49	**-0.07**	**0.27^∗∗^**	**0.26^∗∗^**						
5. Education^c^	1.98	0.89	**0.17^∗^**	-**0.27^∗∗^**	**-0.36^∗^**	**-0.08**					
6. Hindrance stressors	4.04	0.79	**0.00**	0.00	-0.02	**0.04**	**0.03**	(0.86)			
7. Challenge stressors	4.01	0.64	**0.07**	-0.08	-0.08	**0.01**	**0.08**	0.41^∗∗^	(0.87)		
8. Prohibitive voice	4.22	0.61	**0.10**	0.03	0.11^∗^	**0.01**	**0.02**	-0.10^∗^	0.11^∗^	(0.84)	
9. Promotive voice	5.09	0.95	**0.13^∗^**	0.00	-0.02	**0.06**	**0.02**	-0.07	0.11^∗^	0.66^∗∗^	(0.88)

**Internal consistency reliabilities are shown on the diagonal in parentheses, and Spearman’s correlation results are shown in bold. *^*a*^Gender: 0 = “female”; 1 = “male.” ^*b*^Marriage: 1 = “unmarried”; 2 = “married”; 3 = “divorced.” ^*c*^Education: 1 = “high school and bellow”; 2 = “bachelor’s degree”; 3 = “master’s degree”; 4 = “doctorate and above.” ^∗^p < 0.05. ^∗∗^p < 0.01.***

### Descriptive Statistics

The means and standard deviations, internal consistency reliabilities, and correlations (Spearman’s correlation for categorical variables and Pearson’s correlation for the others) among the study variables are shown in Table 2. In addition, the hindrance stressors were significantly and negatively correlated with prohibitive voice (*r* = −0.10, *p* < 0.05) but were not correlated with promotive voice (*r* = −0.07, *p* > 0.05). The challenge stressors were significantly and positively correlated with prohibitive voice (*r* = 0.11, *p* < 0.05) and promotive voice (*r* = 0.11, *p* < 0.05). Thus, this evidence provided preliminary support for our hypotheses. In addition, the Cronbach’s alpha value of every dimension was above 0.70, indicating the high internal consistency and validity of the constructs (Nunnally, 1978).

### Hypothesis Testing

We conducted HLM to examine our hypotheses. Hypotheses 1a and 1b proposed that there would be a curvilinear (U-shaped) relationship between hindrance stressors and prohibitive and promotive voice. We added hindrance stressors in Model 2 and hindrance stressors-squared in Model 3. Tables 3, 4 show that the quadratic term of the hindrance stressors was positively related to prohibitive voice (γ = 0.09, *p* < 0.05) and promotive voice (γ = 0.08, *p* < 0.05) when controlling for the effect of the linear term (γ = −0.13, *p* < 0.01) and (γ = −0.10, *n.s.*). Thus, Hypotheses 1a and 1b were well supported.

**TABLE 3:** The relationship between stressors and prohibitive voice.

**Level and variables**	**Prohibitive voice**						
	**Model 1**	**Model 2**	**Model 3**	**Model 4**	**Model 5**	**Model 6**	**Model 7**
Intercept	4.66^∗∗^	4.62^∗∗^	4.55^∗∗^	4.62^∗∗^	4.63^∗∗^	4.58^∗∗^	4.62^∗∗^
Gender	–0.01	0.02	–0.01	–0.02	–0.02	–0.01	–0.02
Age	0.00	0.01	0.01	0.01	0.01	0.01	0.01
Tenure	0.00	0.00	0.00	0.00	0.00	0.00	0.00
Marriage	–0.02	0.03	0.03	–0.03	–0.03	–0.04	–0.03
Education	0.02	0.02	0.02	0.02	0.02	0.01	0.02
Hindrance stressors		–0.16^∗∗^	–0.13^∗∗^	–0.16^∗∗^	–0.16^∗∗^	−0.10^∗^	−0.17^∗^
Challenge stressors		0.16^∗∗^	0.16^∗∗^	0.16^∗∗^	0.16^∗∗^	0.25^∗∗^	0.16^∗∗^
Hindrance stressors-squared			0.09^∗^			0.11^∗∗^	
Challenge stressors-squared				0.00			0.02
Challenge stressors × hindrance stressors					–0.02	−0.10^∗^	–0.02
Challenge stressors × hindrance stressors-squared						−0.06^∗^	
Hindrance stressors × challenge stressors-squared							0.01
Pseudo *R*^2^	0.24	0.29	0.31	0.29	0.29	0.34	0.29

**There were no organization-level independent variables. Pseudo R^2^ indicates the amount of total variance in the dependent variable explained by predictors in the model. Pseudo R^2^ = 1 − (σ^2^ + τ)/Var (y_*ij*_). ^∗^p < 0.05. ^∗∗^p < 0.01.**

**TABLE 4:** The relationship between stressors and promotive voice.

**Level and variables**	**Promotive voice**						
	**Model 1**	**Model 2**	**Model 3**	**Model 4**	**Model 5**	**Model 6**	**Model 7**
Intercept	4.83^∗∗^	4.80^∗∗^	4.74^∗∗^	4.77^∗∗^	4.81^∗∗^	4.76^∗∗^	4.78^∗∗^
Gender	0.03	0.02	0.03	0.03	0.02	0.03	0.03
Age	0.01	0.01	0.01	0.01	0.01	0.01	0.01
Tenure	–0.01	–0.01	–0.01	–0.01	–0.01	–0.01	–0.01
Marriage	0.09	0.07	0.07	0.07	0.07	0.06	0.07
Education	–0.00	–0.00	–0.01	–0.00	–0.00	–0.01	–0.00
Hindrance stressors		–0.12	–0.10	−0.12^∗^	−0.11^∗^	–0.06	–0.13
Challenge stressors		0.15^∗^	0.15^∗^	0.16^∗∗^	0.15^∗^	0.23^∗∗^	0.16^∗^
Hindrance stressors-squared			0.08^∗^			0.09^∗^	
Challenge stressors-squared				0.04			0.06
Challenge stressors × hindrance stressors					–0.01	–0.08	–0.04
Challenge stressors × hindrance stressors-squared						−0.06^∗^	
Hindrance stressors × challenge stressors-squared							0.01
Pseudo *R*^2^	0.12	0.15	0.16	0.15	0.15	0.17	0.15

**There were no organization-level independent variables. Pseudo R^2^ indicates the amount of total variance in the dependent variable explained by predictors in the model. Pseudo R^2^ = 1 − (σ^2^ + τ)/Var (y_*ij*_). ^∗^p < 0.05. ^∗∗^p < 0.01.**

Hypotheses 2a and 2b proposed that challenge stressors were linearly positively correlated with prohibitive voice and promotive voice. Following the same procedures described above, we added challenge stressors in Model 2 and challenge stressors-squared in Model 4. Tables 3, 4 show that the effect of the challenge stressors-squared on prohibitive voice (γ = 0.00, *n.s.*) and promotive voice (γ = −0.04, *n.s.*) was not significant, while the positive effect of the linear term on prohibitive voice (γ = 0.16, *p* < 0.01) and promotive voice (γ = 0.16, *p* < 0.01) was significant when controlling for the effect of the hindrance stressors. Thus, Hypotheses 2a and 2b were well supported.

Hypothesis 3a predicted that hindrance stressors have a negative relationship with prohibitive voice when challenge stressors are high (Hypothesis 3a-i) and a U-shaped relationship with prohibitive voice when challenge stressors are low (Hypothesis 3a-ii). As shown in Table 3, the quadratic-by-linear interaction term between hindrance stressors and challenge stressors (Model 6) was negatively related to prohibitive voice (γ = −0.06, *p* < 0.05). But Model 7 showed that the quadratic-by-linear interaction term between challenge stressors and hindrance stressors was not significantly related to prohibitive voice (γ = 0.01, *n.s.*). Hypothesis 3b predicted that hindrance stressors have a negative relationship with promotive voice when challenge stressors are high (Hypothesis 3b-i) and a U-shaped relationship with prohibitive voice when challenge stressors are low (Hypothesis 3b-ii). Similarly, Table 4 shows that the quadratic-by-linear interaction term between hindrance stressors and challenge stressors (Model 6) was negatively related to promotive voice (γ = −0.06, *p* < 0.05). However, Model 7 shows that the quadratic-by-linear interaction term between challenge stressors and hindrance stressors was not significantly related to promotive voice (γ = 0.01, *n.s.*), which indicated that challenge stressors significantly moderate the relationship between hindrance stressors-squared and prohibitive voice and promotive voice. Hypotheses 3a and 3b were well supported.

**FIGURE 2:**
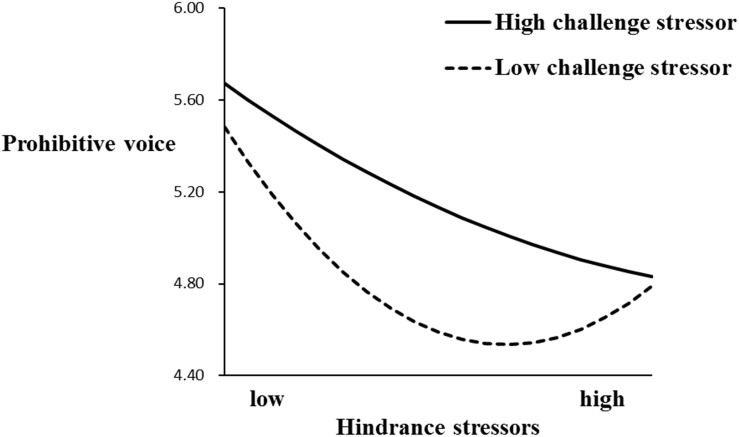
Moderating effect of challenge stressors on the relationship between hindrance stressors and prohibitive voice.

**FIGURE 3:**
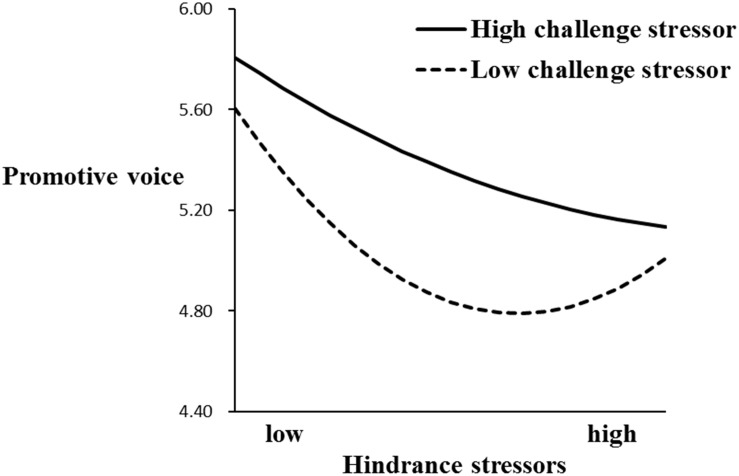
Moderating effect of challenge stressors on the relationship between hindrance stressors and promotive voice.

To further analyze the quadratic-by-linear interaction effect, we tested the simple slopes of the U-shaped line corresponding to low (*mean* - 1*SD*) and high (*mean* + 1*SD*) levels of challenge stressors (Aiken and West, 1991). According to Table 5 and Figure 2, under the condition of high challenge stressors, the results indicated that the relationship between hindrance stressors and prohibitive voice was linearly negative as reflected by a significant coefficient for hindrance stressors-squared (γ = −0.19, *p* < 0.01). Conversely, under the condition of low challenge stressors, the relationship between hindrance stressors and prohibitive voice was U-shaped as reflected by the significant coefficient for hindrance stressors-squared (γ = 0.17, *p* < 0.01). Similarly, according to Table 5 and Figure 3, the relationship between hindrance stressors and promotive voice was significantly linearly negative as reflected by a significant coefficient for hindrance stressors-squared (γ = −0.14, *p* < 0.05). Conversely, under the condition of low challenge stressors, the relationship between hindrance stressors and promotive voice was U-shaped as reflected by the significant coefficient for hindrance stressors-squared (γ = 0.15, *p* < 0.01). Hypotheses 3a and 3b are well supported.

**TABLE 5:** The effect of job stressors on prohibitive and promotive voice.

**Level and variables**	**Prohibitive voice**			**Promotive voice**		
	**Estimate**	**SE**	**95% CI**	**Estimate**	**SE**	**95% CI**
When challenge stressors are high						
Hindrance stressors	−0.19^∗∗^	0.05	[−0.29, −0.10]	−0.14^∗^	0.07	[−0.27, −0.01]
Hindrance stressors-squared	0.04	0.05	[−0.05, 0.14]	0.03	0.06	[−0.09, 0.15]
When challenge stressors are low						
Hindrance stressors	0.00	0.07	[−0.14, 0.14]	0.01	0.08	[−0.14, 0.17]
Hindrance stressors-squared	0.17^∗∗^	0.05	[0.08, 0.26]	0.15^∗^	0.05	[0.06, 0.23]

**^∗^p < 0.05. ^∗∗^p < 0.01.**

## Discussion

According to the COR theory, we presented evidence of a U-shaped relationship between hindrance stressors and employees’ prohibitive and promotive voice. The results also showed that challenge stressors were positively related to promotive and prohibitive voice and moderated the U-shaped relationship between hindrance stressors and voice behavior. The relationship between hindrance stressors and prohibitive and promotive voice is (i) linearly negative when challenge stressors are high but (ii) U-shaped when challenge stressors are low.

Our research has some theoretical implications. First, we extend the previous research on the challenge-hindrance framework to propose a curvilinear relationship that concludes simultaneously hindrance stressors’ positive and negative effects on voice (Tangirala and Ramanujam, 2008). We also validate the positive effect of challenge stressors on voice behavior. More importantly, we extend the prior research to prove that the moderation effect of challenge stressors on the U-shaped relationship would alleviate employees from reaching the hindrance stressor inflection point (Hollebeek and Haar, 2012). Second, our results enrich the theoretical and empirical foundations of the voice literature by examining the relationship between job stressors and both promotive and prohibitive voice (Hu et al., 2018). We find that relationships between hindrance stressors and prohibitive and promotive voice are U-shaped. We indicate that individuals choose prohibitive and promotive voice to prevent resource losses when they are confronted with high levels of hindrance stressors. Third, our study extends the application of the COR theory by demonstrating the relationship between the challenge-hindrance framework and voice behavior. Despite prominent studies focused on the resource-conservation motivation of voice under stressors (Hu et al., 2018), we reveal that a high level of hindrance stressors triggers resource-acquisition motivation to speak up. Thus, our study deepens the understanding of the role of stressors in the COR theory and voice behavior as a response to resource losses (Qin et al., 2014). We also move beyond the limitation of the previous studies by considering the non-linear relationship between hindrance stressors and voice behavior.

Our results also have several important practical implications. First, these results reveal the predictors that were found to be relevant for voice. The downward slope of our model indicates that managers can promote voice behavior by reducing hindrance stressors, but it is not advocated to increase the level of hindrance stressors. For example, job design and workload should be clearly set based on employees’ practical working capability, and employees should set clear role boundaries to avoid role ambiguity. In addition, challenge stressors lead to more efficient and committed employees who exhibit voice behavior. Similarly, the development of greater chances for responsibility and challenging roles at workshops might enhance positive effects of challenge stressors on voice behavior. Managers should try to equilibrate such balance, foster social support, etc. Second, our results can help practitioners and organizations recognize the job stressors’ level related to voice and optimal functioning. Either a low or high level of hindrance stressors can activate employees’ high level of voice behavior. Therefore, it is vital to recognize the true hindrance stressors’ level when employees choose to enact voice behavior. It may be naïve to expect that all employees will always have low levels of hindrance stressors. It is necessary to recognize that hindrance stressors among employees likely signal other organizational problems and offer an opportunity for future improvements. In particular, employees need more attention when workers’ resource-acquisition motivation is activated. Because workers who confront a high level of hindrance stressors have more intimate knowledge and information about these stressors, they can identify the potential problems or failures of the existing organizational practices (Frieder et al., 2015). On the other hand, employees are unlikely to speak up when they face a medium level of hindrance stressors. This reflects the peak of strain and indicates exhaustion, which requires additional attention for employees (Qin et al., 2014). Therefore, the regular monitoring of the prevailing job stressors and resources as well as of voice behavior is essential for recognizing possible problems that require action. Third, given their potential positive gains, challenge stressors alleviate the negative effect of hindrance stressors, which suggests some relative factors for hindering employees from expressing their concerns and speaking up. For workers, high levels of challenge stressors will significantly influence the hindrance stressors’ effect, with high levels of challenge stressors leading to slower increases in resource exhaustion and then employee voice behavior when confronted with high levels of hindrance stressors. Thus, managers should distinguish and deal with each stressor separately (Antwi et al., 2019). Organizations could also provide training courses to enhance employees’ practical skills to cope with stressors, or create more opportunities for employees to receive psychological counseling and consultation (Hollebeek and Haar, 2012).

### Limitations and Future Studies

This study has several limitations. First, our study applied a cross-sectional design to examine the effect of job stressors on voice behavior. Therefore, it would be premature to draw exact conclusions about causality. Future studies could adopt longitudinal approaches to address the trend of those changes. Second, although this study made an important contribution by examining the relationship between job stressors and voice behavior, future research should also explicitly measure the potential mechanisms in our model to directly examine their roles in the association between job stressors and voice behavior. Third, we did not consider confounders well in the study design, and future research should simultaneously investigate the team psychological safety climate, which has been regarded as a factor influencing employee voice behavior (Wei et al., 2015). Fourth, we regarded both stressors as individual-level variance in this research, which might neglect the possibility of both stressors as between-level variables. More empirical research is needed to examine the possibility and its influence on the estimation of the relationship between stressors and voice behavior. Finally, for the differences between the structure and functioning of Chinese and Western organizations, individualism is a priority in Western society, but interpersonal harmony and collective value are priorities in traditional Chinese culture (Qin et al., 2014). Future research should extend the sample to multinational companies to explore the issue of cross-cultural research in stressors and voice behavior.

## Conclusion

Following the COR theory, we examined a U-shaped relationship between hindrance stressors and voice behavior. We concluded that employees decrease any voice behavior when they are confronted with a low level of hindrance stressors for resource-reservation motivation. However, employees must enact prohibitive and promotive voice for resource-acquisition motivation when confronted with a high level of hindrance stressors. Moreover, we proved that the combined effect of challenge stressors would buffer the negative effect of hindrance stressors and prevent employees from speaking up in the face of a high level of hindrance stressors. Employees with high challenge stressors are still motivated by resource-reservation motivation and are reluctant to enact voice behavior. Therefore, we highlighted the utility of the COR theory for understanding the U-shaped relationships between hindrance stressors and voice behavior, and proposed practical implications for organizations to elevate employees’ voice behavior.

## Data Availability Statement

The data collected for this study are available on [OSF] [Zhou, L. (2019, August 11). voice behavior1. Retrieved from https://osf.io/uqacj/].

## Ethics Statement

An ethics approval was not required as per institutional guidelines and national laws and regulations because no unethical behaviors existed in this study. We just conducted paper–pencil test and were exempt from further ethics board approval since our study did not involve human clinical trials or animal experiments. In the survey process, all participants were informed that participation was voluntary and assured that their responses would be only used for our research and kept confidential strictly. Therefore, only those who were willing to participate were recruited. In other words, the informed consent of the participants was implied through survey completion. To ensure confidentiality, the questionnaires completed during their working hours were directly returned to these research assistants in sealed envelopes.

## Author Contributions

LZ and ZL designed and drafted the work. LZ and KY collected the data. LZ analyzed the data and drafted the manuscript. ZW critically revised the manuscript. All authors gave the final approval of the manuscript before the submission.

## Conflict of Interest

The authors declare that the research was conducted in the absence of any commercial or financial relationships that could be construed as a potential conflict of interest.
